# Effect of young age (below 40 years) on oncologic outcomes in Lebanese patients with breast cancer: a matched cohort study

**DOI:** 10.1186/s12885-024-11910-w

**Published:** 2024-05-04

**Authors:** Eman Sbaity, Hani Tamim, Ghada El-Hajj Fuleihan, Jaber Abbas, Mariam Zahwe, Razan El Sayed, Ali Shamseddine

**Affiliations:** 1 Department of Surgery, American University of Beirut Medical Center, P.O. Box: 11-0236, Riad El-Solh, Beirut, 1107 2020 Lebanon; 2 Department of Internal Medicine, American University of Beirut Medical Center, Beirut, Lebanon; 3 Calcium Metabolism and Osteoporosis Program, American University of Beirut, Beirut, Lebanon; 4 Scholars in HeAlth Research Program (SHARP), American University of Beirut Medical Center, Beirut, Lebanon; 5 Faculty of Medicine, American University of Beirut, Beirut, Lebanon; 6 Center for Translational Injury Research, Department of Surgery, McGovern Medical School, University of Texas Health Science Center (UT Health), Houston, USA; 7 Department of Hematology/Oncology, American University of Beirut Medical Center, Beirut, Lebanon

**Keywords:** Breast cancer, Young age, Prognosis, Survival, Recurrence

## Abstract

**Background:**

Developing countries have a significantly higher incidence of breast cancer in patients younger than 40 years as compared to developed countries. This study aimed to examine if young age at diagnosis is an independent prognostic factor for worse survival outcomes in breast cancer as well as the effect of age on Disease-free survival (DFS) and local recurrence free survival (LRFS) after adjusting for various tumor characteristics, local and systemic treatments.

**Methods:**

This is a secondary analysis of prospective cohort of patients from two existing databases. We identified patients with breast cancer aged 40 years or less and we matched them to those older than 40 years. We also matched based on stage and molecular subtypes. In cohort 1, we matched at a ratio of 1:1, while in cohort 2 we matched at a ratio of 1:3.

**Results:**

In cohort 1, Disease-free survival (DFS) at 5 years was significantly shorter for those younger than 40 years (75.6% and 92.7% respectively; *p* < 0.03). On multivariate analysis, only chemotherapy was found to be significant, while age was not found to be an independent predictor of prognosis. Local recurrence free survival at 5 years was similar between both age categories. Only hormonal therapy is a significant predictor for LRFS at 5 years. In the second cohort, DFS and LRFS at 3 years were similar between those younger and those older than 40 years. On multivariate analysis, no factor including age was found to be an independent predictor of prognosis.

**Conclusion:**

Data in the literature is controversial on the effect of young age on breast cancer prognosis. Our findings could not demonstrate that age is an independent prognostic factor in our population. There is a need for outcomes from larger, prospective series that have longer follow-ups and more data from our region.

## Background

Breast cancer is the most common cancer in females globally, with 2.3 million women diagnosed with breast cancer in 2020 [1]. Median age of breast cancer varies between developed countries of 41.9 years [2] compared to 44–48 years in developing countries [3, 4]. As for Lebanon, the median age is 49.8 years [5].

Although breast cancer does not commonly occur in patients younger than 40 years of age, it is a leading cause of death from cancer in this young population. The proportion of young patients diagnosed with breast cancer is much higher in developing countries, 19-33.3% in Arab countries [6–9] compared to developed countries, 5–7% in the United States of America. This may be explained by different population pyramid, environmental or genetic factors [10].

Reports of breast cancers in young patients show higher proportions of adverse clinic-pathologic features, Her2 neu expression, Estrogen receptors (ER)- and progesterone receptor (PR)-negative tumors, and high-grade tumors that tend to be larger and to involve regional lymph nodes [11–13]. In addition, authors documented a different distribution of molecular breast cancer subtypes between young breast cancer and older population [14].

The effect of young age on oncologic outcomes is controversial in the literature. Initially, physicians attributed the worse prognosis of breast cancer at a younger age to an advanced stage of presentation, adverse pathologic subtypes, and less aggressive treatments [15–18]. This is supported by results from a large series of patients showing young age to be an independent risk factor for worse disease-free survival (DFS), Distant Disease-Free Survival (DDFS), and overall survival (OS) [18, 19]. Other authors consider young age as a surrogate marker of more advanced stage or more aggressive phenotypes resulting in worse prognosis.

Due to the perception of a more aggressive disease, young breast cancer patients frequently receive total mastectomies. Thus, it is of high clinical importance to understand whether breast cancer surgery (BCS) is associated with a higher risk of local recurrence to better counsel young patients. Many large retrospective series showed a higher rate of local recurrence in BCS as compared to total mastectomy performed at a young age, but no difference in survival [18, 20–25]. Thus, when indicated, BCS is still a viable option for young patients..

The aim of this cohort study is to examine if young age at diagnosis is an independent prognostic factor for worse survival outcomes in breast cancer as well as the effect of age on DFS and local recurrence free survival (LRFS) after adjusting for various tumor characteristics, local and systemic treatments received.

## Methodology

### Study design

We conducted a secondary analysis of two existing prospective cohorts of breast cancer patients. We matched patients younger than 40 years to those older than 40 years at a ratio of 1:3. We matched cases based on the stage of breast cancer at presentation and molecular subtypes.

### Data sources

The study is a secondary analysis of two existing databases. The first is prospectively collected data of all Lebanese non-metastatic breast cancer patients who received any part of their treatment at the American University of Beirut Medical Center (AUBMC) between the years 2011–2014 to study the difference in outcomes between the two age groups (IRB study #IM.AS.17). It includes 123 Lebanese patients (with 47 patients below the age of 40 years). The second is an IRB-approved prospectively collected database of the clinical research unit at Basile Cancer Institute at AUBMC (BIO-2018-0302), including all consecutive breast cancer patients who have presented to AUBMC from October 2014 to December 2016. Informed consent to participate in the initial studies was obtained from all participants. We performed a comparison between both datasets regarding tumor characteristics and received treatments. This revealed statistically significant differences in both populations, so we decided to analyze each cohort separately.

### Eligibility criteria

We included patients with:

- Non-metastatic biopsy-proven breast cancer.
- Lebanese women, older than 18 years.
- Received any part of their treatment or followed up at AUBMC.

We excluded:

- Male breast cancer patients.
- Patients with unclassified tumor.
- Patients with missing data on stage or ER, PR, or Her2 NEU or staging information.

### Sampling Frame

At our institution, breast cancer patients are treated by dedicated breast surgical oncologists, breast medical oncologists, and breast radiation oncologists, ensuring similar and up-to-date treatment plans based on National Comprehensive Cancer Network (NCCN) guidelines, American Society of Cancer Oncology (ASCO), and ASBS (American Society of Breast Surgery). In addition, most of these patients are discussed in the weekly Breast Tumor Board before initiation of treatments.

### Outcome measures

Primary outcome:

- DFS at 3 years, defined by survival without clinical or radiological evidence of recurrence of disease, whether local, distant, or both.

Secondary outcomes:

- Local recurrence Free Survival at 3 years (LRFS) defined by clinical or radiological evidence of disease in the affected breast or regional nodal basin.
- Overall Survival (OS) at 3 years defined from time of diagnosis till date of death or last follow up.
- Distant Metastasis Free Survival (DMFS) is defined from the date of diagnosis till the date of distant metastasis or till the date of last follow up in patients who did not experience metastasis.
- Effect of various tumor grade, local and systemic treatments received on DFS, LRFS, and DMFS.

### Statistical analysis

We conducted all analyses using SPSS Version 24, and statistical significance was assumed at a *p* < 0.05. Prognostic factors were compared between the two groups using chi-square test for categorical variables and independent t-test for continuous variables. We estimated DFS, LRFS, DMFS, and OS, using Kaplan–Meier method and Log-Rank test for the different survival curves between both age categories. We then performed a univariate analysis comparing the effect of each grade of tumor, chemotherapy, Hormonal therapy, Herceptin, type of surgery, radiation therapy, and BMI on DFS, LRFS, and DMFS at three years follow up. We used Cox regression analysis to assess how survival outcomes change between both age groups after controlling for grade of tumor, chemotherapy, hormonal therapy, Herceptin therapy, surgery type, and radiation therapy. COX Regression was done using the forward and backward methods.

## Results

### Cohort 1

#### Description

The first cohort included 122 breast cancer patients, 77 patients above the age of 40 years, and 45 patients below or equal to 40 years. We performed 1:1 matching, where 41 breast cancer patients aged 40 or younger were matched to 41 breast cancer patients older than 40. The patients’ molecular subtypes and stages are shown in Table 1.

**Table 1:** Matched Cohort 1 comparison

Cohort 1		Above 40 years *n* = 41 (%)	Below or equal to 40 years *n* = 41 (%)	Total *n* = 82(%)	*P* value
**Stage**	**Stage I**	10(24.4)	10(24.4)	20(24.4)	1
	**Stage II**	22(53.7)	22(53.7)	44(53.7)	
	**Stage III**	9(22)	9(22)	18(22)	
**Molecular Subtype**	**Luminal A**	17(41.5)	17(41.5)	34(41.5)	1
	**Luminal B**	12(29.3)	12(29.3)	24(29.3)	
	**TN**	10(24.4)	10(24.4)	20(24.4)	
	**HER 2 Neu**	2(4.9)	2(4.9)	4(4.9)	
**Grade**	**1**	4(9.8)	7(17.1)	11(13.4)	0.276
	**2**	14(34.1)	18(43.9)	32(39)	
	**3**	23(56.1)	16(39)	39(47.6)	
**BMI**	**Underweight**	1(2.4)	1(2.4)	2(2.4)	0.455
	**Normal**	16(39)	23(56.1)	39(47.6)	
	**Overweight**	14(34.1)	11(26.8)	25(30.5)	
	**Obese**	10(24.4)	6(14.6)	16(19.5)	
**Family history**	**Yes**	17(41.5)	20(48.8)	37(45.1)	0.506
	**No**	24(58.5)	21(51.2)	45(54.9)	
**Surgery type**	**PM**	28(68.3)	18(43.9)	46(56.1)	0.026
	**TM**	13(31.7)	23(56.1)	36(43.9)	
**Radiotherapy**	**Yes**	32(84.2)	29(80.6)	61(82.4)	0.680
**Chemotherapy**	**Yes**	28(68.3)	27(69.2)	55(68.8)	0.928
**Trastuzumab**	**Yes**	5(12.2)	12(30)	17(21)	0.049
**Hormonal Therapy**	**Yes**	31(75.6)	34(85)	65(80.2)	0.289

#### Comparison of treatments received

Total mastectomy was performed only in onethird of patients above 40 years as compared to 56% of patients below 40 years with *p* = 0.026. Radiotherapy and chemotherapy were administered in a similar proportion for both groups without any statistical difference. A higher proportion of patients in the younger subgroup received trastuzumab as compared to the older subgroup, 30% and 12.2% respectively (*p* = 0.049). Similarly, patients in the younger subgroup are more likely to receive hormonal therapy, but without reaching a statistical significance. (Table 1)

#### Outcomes

Only one death is documented in the older subgroup, and two deaths in the younger subgroup. Local recurrence is double in the younger age group, with 6 local recurrence events (14.6%) compared to 3 local recurrence events (7.3%) in the older age group. Distant metastasis occurred more in the younger age groupin 9 patients (22%) compared to 6 (14.6%) in the older age group.

#### Survival analysis

- **DFS at 5 years**: in young patients, the DFS is 75.6% compared to 92.7% in older patients with *p* = 0.035. In patients who received chemotherapy, DFS is lower in the younger age group,74.1% compared to 100% in those older than 40 years, with a statistically significant *p*-value of 0.005. Similarly, in patients who received hormonal therapy, DFS is lower in the younger subgroup of patients, at76.5% compared to 96.8%, with a *p*-value of 0.016. On multivariate analysis, only chemotherapy was an independent prognostic factor for DFS at 5 years. Age was not found to be an independent prognostic factor for DFS. (Table 2)
- **LRFS at 5 years**: among those above 40 years, the is 92.7% (3 local recurrence events) and 87.8% (5 local recurrence events) among those below or equal to 40 years (*p* = 0.361).
- We did not find any clinically or statistically significant difference when stratifying LRFS at 5 years according to the different loco-regional and systemic therapies. Patients who underwent partial mastectomy have an LRFS of 96.4% in the older group and 94.4% in the younger group, with no statistical significance (*p* = 0.655). For those who underwent total mastectomy, LRFS is 84.6% and 82.6% in the older and younger group, respectively (*p* = 0.821). In multivariate cox regression analysis, only hormonal therapy was found to be a predictor for worse LRFS at 5 years, while age was not found to be an independent prognostic factor.
- **OS at 5 years**: in the above 40 years group, the overall survival at 5 years is 97.6% and 95.1% in the below or equal to 40 years group, with no statistical significance (*p* = 0.490) (Table 3).

**Table 2:** Stratified DFS at 5 years for Matched Cohort 1

		Above 40 years (*n* = 41)	Below or equal to 40 years (*n* = 41)	Total (*n* = 82)	*P* value
**DFS 5 years**		92.7%	75.6%	84.1%	0.032
**Stratified DFS at 5 years**					
**Grade**	**1**	100%	85.7%	90.9%	0.371
	**2**	100%	72.2%	84.4%	0.038
	**3**	87%	75%	82.1%	0.380
**BMI**	**Underweight**	100%	100%	100%	
	**Normal**	93.3%	73.9%	82.1%	0.120
	**Overweight**	92.9%	81.8%	88.0%	0.432
	**Obese**	90%	66.7%	81.3%	0.216
**Family History**	**Yes**	100%	80%	89.2%	0.191
	**No**	87%	71.4%	80%	0.045
**Surgery type**	**PM**	96.4%	88.9%	93.5%	0.280
	**TM**	84.6%	65.2%	72.2%	0.261
**Radiotherapy**	**Yes**	93.8%	79.3%	86.9%	0.093
	**No**	83.3%	71.4%	76.9%	0.713
**Chemotherapy**	**Yes**	100%	74.1%	87.3%	0.005
	**No**	76.9%	75%	76%	0.700
**Trastuzumab**	**Yes**	100%	66.7%	76.5%	0.158
	**No**	91.7%	78.6%	85.9%	0.123
**Hormonal Therapy**	**Yes**	96.8%	76.5%	86.2%	0.016
	**No**	80%	66.7%	75%	0.549

**Table 3:** Matched Cohort 1 Survival Outcomes at 5 years

	Above 40 years (*n* = 41)	Below or equal to 40 years (*n* = 41)	Total (*n* = 82)	*P* value
**DFS 5 years**	92.7%	75.6%	84.1%	0.035
**LRFS 5 years**	92.7%	87.8%	90.2%	0.361
**OS 5 years**	97.6%	95.1%	96.3%	0.490

### Cohort 2

#### Description

There are a total of 399 patients in cohort 2, with 55 breast cancer patients aged 40 or younger matched to 165 breast cancer patients older than 40 years, at a 1:3 ratio. In the older age group, a total of 40 patients (28%) had grade 3 breast cancer, compared to 22 patients (44%) in the younger age category with a significant *p*-value of 0.03 (Table 4).

**Table 4:** Matched Cohort 2 comparison between younger and older patients

Cohort 2		Above 40 years *n* = 165(%)	Below or equal to 40 years *n* = 55(%)	Total *n* = 220(%)	*P* value
**Stage**	**Stage I**	54(32.7)	18(32.7)	72(32.7)	0.995
	**Stage II**	62(37.6)	21(38.2)	83(37.7)	
	**Stage III**	49(29.7)	16(29.1)	65(29.5)	
**Molecular Subtype**	**Luminal A**	77(46.7)	26(47.3)	103(46.8)	0.999
	**Luminal B**	72(43.6)	24(43.6)	96(43.6)	
	**TN**	13(7.9)	4(7.3)	17(7.7)	
	**HER 2 Neu**	3(1.8)	1(1.8)	4(1.8)	
**Grade**	**1**	32(20.9)	11(21.6)	43(21.1)	0.030
	**2**	80(52.3)	17(33.3)	97(47.5)	
	**3**	41(26.8)	23(45.1)	64(31.4)	
**BMI**	**Underweight**	-	-	-	0.179
	**Normal weight**	46(29.7)	19(37.3)	65(31.6)	
	**Overweight**	54(34.8)	21(41.2)	75(36.4)	
	**Obese**	55(35.5)	11(21.6)	66(32)	
**Family History**	**Yes**	46(29.3)	22(42.3)	68(32.5)	0.083
	**No**	111(70.7)	30(57.7)	141(67.5)	
**Surgery type**	**PM**	87(53.4)	34(63)	121(55.8)	0.219
	**TM**	76(46.6)	20(37)	96(44.2)	
**Radiotherapy**	**Yes**	111(67.7)	35(64.8)	146(67)	0.698
**Chemotherapy**	**Yes**	119(74.4)	41(77.4)	160(75.1)	0.663
**Trastuzumab**	**Yes**	21(12.7)	10(18.2)	31(14.1)	0.314
**Hormonal Therapy**	**Yes**	101(69.7)	39(81.3)	140(72.5)	0.119

#### Comparison of treatments received

Mastectomy was performed more frequently in the older group, but the difference is not statistically significant ;76 (46.6%) patients above 40 years underwent total mastectomy as compared to 20 (37%) patients below 40 years (*p* = 0.219). For radiation therapy, 67.7% of patients over 40 years received radiotherapy, and 64.8% of patients in the younger group (*P* = 0.698). Both age subgroups received chemotherapy in a similar proportion, with 74.4% of patients in the above 40 years groupand 77.4% in the below or equal to 40 years group (*p*-value of 0.663). A higher proportion of patients in the younger subgroup received trastuzumab and hormonal therapy as compared to the older subgroup but without reaching statistical significance. A total of 81.3% of patients younger than 40 years received hormonal therapy compared to 69.7% of patients older than 40 years (*p* = 0.119).

#### Outcomes

No deaths are documented in any subgroup. Local recurrence did not occur in the younger group, and only 1 event (0.6%) occurred in the older group. Distant metastasis occurred in 1 patient (1.8%) in the below 40 years subgroup and in 6 patients (3.6%) in the older age group.

#### Survival analysis

- **DFS at 3-years**: is slightly shorter for the younger age patient category but without reaching statistical significance (97% versus 98.2%; *p* = 0.621). DFS is 100% for partial mastectomy in the younger group and 97.7% (2 events) in the older group (*p* = 0.346). Among patients treated with total mastectomy, DFS is 97.4% in the older group and 95% in the younger group, with no statistical significance (*p* = 0.645). On multivariate Cox regression analysis for DFS at 3 years for cohort 2, no factor was found to be a significant predictor of survival.
- **LRFS at 3 years**: local recurrence occurred only in 1 patient (99.4%) in the younger group and none in the older group. (*p* = 0.537). On multivariate Cox regression analysis for cohort 2, no factor was found to be a significant predictor of survival for LRFS at 3 years.

## Discussion

In our first cohort, the distribution of early and advanced breast cancer was similar. In the second cohort, older breast cancer patients were more likely to be present in the early stages. This result was similar to that reported by many authors who mentioned that young patients with breast cancer present with a more aggressive clinical picture and advanced stage as compared to older patients. Cancers in the younger age group are usually detected by the patients themselves and,consequently, are often bigger in size and more advanced than the screen-detected tumors in those above 40 years [26, 27].

We had a similar proportion of molecular subtypes in both age categories, with a slightly higher proportion of Triple negative molecular subtype in our young patient population. This is different from the proportions known for breast cancer patients. The literature reports on a difference in the distribution of breast cancer molecular subtypes between younger and older patients [14, 28]. Variability in molecular subtyping among different populations may be due to variability in pathology reviews and different definitions to categorize the 4 subtypes, where mainly the confusion happens with deciding on luminal A and B.

Age was not found to be an independent prognostic factor in our cohort of patients, which is matched for stage and molecular subtype and after adjusting for treatments received. DFS after 5 years was statistically lower in the younger group. However, LRFS at 3 years and 5 years were not statistically different between both age categories. Nixon et al. reported similar results where young breast cancer has a higher local recurrence rate and inferior DFS [12, 13, 29–37]. Recurrence events in breast cancer usually happen at a median of 32 months, which falls within our follow-up period. Nevertheless, a longer follow-up is required to make a solid conclusion.

When we stratified the outcomes according to the type of surgery done, there was no significant difference in the DFS at 3 and 5 years and LRFS at 3 years in both age categories. This result was similar to the National Cancer Institute randomized study that showed similar local recurrence between both arms of treatment for BCS and total mastectomy [38, 39]. Age was not found to be a predictor of local recurrence when total mastectomy was performed. However, in the Institut Gustave-Roussy Breast Cancer Group Distribution of local recurrence based on age grouping of less than and more than 40 years, there was a three times higher rate of local recurrence in the younger patients with BCS.

On univariate analysis of cohort 1, patients receiving radiotherapy and those below 40 years had lower 5-year DFS, but this difference was not statistically significant (*p* = 0.093). We cannot compare our results to the literature because the majority of studies did not report on the use of radiation therapy after BCS, so we cannot identify those local recurrences secondary to lack of radiation. Similarly, it is not possible to discern the effect of post mastectomy radiation on preventing local recurrences.

In the strata of patients who received chemotherapy, DFS is lower in the younger age group, 74.1% compared to 100%, in those older than 40 years, with a statistically significant *p*-value. On multivariate analysis, only chemotherapy was an independent prognostic factor for DFS at 5 years. Age was not found to be an independent prognostic factor for DFS. Similarly, we did not find a statistically significant difference when we stratified LRFS at 5 years for the chemotherapy treatments. On the contrary, the Beadle et al. study [40] showed that local therapies did not affect the rate of local recurrence in young patients, but systemic therapy did affect it with statistical significance. This speaks about a different biology in young patients, where perhaps even in stage I, chemotherapy should be given to improve oncologic outcomes.

In the strata of patients in cohort 1, patients who received hormonal therapy had statistically significant lower DFS at 5 years in the younger subgroup of patients but not for LRFS at 5 years. On multivariate Cox regression analysis, only hormonal therapy was found to be a predictor for worse LRFS at 5 years, while age was not found to be an independent prognostic factor. ER-positive is reported in the literature to be associated with worse prognosis in the younger population. Some attribute this to the fact that young patients were not treated with hormonal therapy until recently. Consequently, the worse prognosis observed in the young population of ER-positive cancermay be due to the differential use of Tamoxifen. In addition, even after treating young breast cancer patients with Tamoxifen, there is high non-compliance. This was demonstrated by a systematic review by Murphy et al. [41].

Among those who received trastuzumab, LRFS is 100% in the older group and 83.3% in the younger group (*p* = 0.315). For those who did not receive trastuzumab, LRFS is similar, with 91.7% in the older group and 89.3% in the younger group, with no statistical significance (*p* = 0.629). Many studies that explored local recurrence in a large cohort of breast cancer patients included patients from the era before the introduction of trastuzumab in the treatment of Her 2 positive breast cancer. This is a major limitation of such studies because trastuzumab dramatically improved in oncologic outcomes when indicated.

### Strengths and limitations

This study aimed to fill a gap in the literature where there is limited data from the modern era of effective surgery, chemotherapy, hormonal, and radiation therapy in young patients. The reported data on the effect of age on breast cancer outcomes is widely retrospective; our data is prospectively collected. Another strength of our study is matching the two age cohorts on the two most important baseline prognostic factors (stage and molecular phenotype). Data on local recurrence after BCT in young patients mainly camefrom series collected over long periods of time and did not receive the modern modalities of breast cancer treatments. Our cohort of patients is a modern cohort treated based on modern therapies.

Because there is no breast cancer national comprehensive database, we could not compare our cohort characteristics to national and thus decide on the generalizability of our results. We have a limited follow-up period for our patients, where the majority reach only 3 years of follow-up.

## Data Availability

The datasets analyzed in the current study are available on request from the corresponding author.

## Abbreviations

ER: Estrogen Receptors
PR: Progesterone Receptor
DFS: Disease Free Survival
DDFS: Distant Disease-Free Survival
OS: Overall Survival
BCS: Breast Cancer Surgery
LRFS: Local Recurrence Free Survival
AUBMC: American University of Beirut Medical Center
Her2: Human Epidermal Growth Factor Receptor 2
NCCN: National Comprehensive Cancer Network
ASCO: American Society of cancer Oncology
ASBS: American Society of Breast Surgery
DMFS: Distant Metastasis Free Survival
BCT: Breast-conserving therapy
